# Baseline socioeconomic status predicting post-COVID-19 symptoms: Results from Isfahan COVID Cohort (ICC) study

**DOI:** 10.1016/j.pmedr.2024.102814

**Published:** 2024-07-07

**Authors:** Mehrdad Rabiee Rad, Mehdi Abbasi, Emad Salimian, Matin Norouzi, Ali Emamjomeh, Fahimeh Haghighatdoost, Shirin Mahmoudi, Jamshid Najafian, Soraya Masoudi, Ghazal Ghasempour Dabaghi, Noushin Mohammadifard, Nizal Sarrafzadegan

**Affiliations:** a Interventional Cardiology Research Center, Cardiovascular Research Institute, Isfahan University of Medical Sciences, Isfahan, Iran; b Heart Failure Research Center, Cardiovascular Research Institute, Isfahan University of Medical Sciences, Isfahan, Iran; c Isfahan Cardiovascular Research Center, Cardiovascular Research Institute, Isfahan University of Medical Sciences, Isfahan, Iran

**Keywords:** Socioeconomic status, COVID-19, Post-acute COVID-19 syndrome, Long haul COVID-19

## Abstract

**Objectives:**

Patients experience complications following their recovery from COVID-19, known as post-COVID-19. This study aimed to investigate the association between baseline socioeconomic status (SES) and post-COVID-19 symptoms among hospitalized and non-hospitalized patients.

**Methods:**

In the study, we used data from the Isfahan COVID Cohort (ICC) study, a 5-year longitudinal cohort study started from March 10, 2020. SES was measured using short form of SES questionnaire (SES-SQ), consisting of employment status, house room number, the number of trips taken, and using notebooks, laptop, or tablet in the house. Cox proportional hazard analysis was used to examine the association between baseline SES and post-COVID-19 symptoms including general, cardiovascular and respiratory systems, adjusting for potential confounders.

**Results:**

Out of the 3912 patients included in the study, 66.4 % reported post-COVID-19 symptoms. There was an association between low SES and increased risk of post-COVID-19 cardiovascular symptoms in the whole population (HR = 1.15; 95 CI, 1.01–1.31, p = 0.039). Considering the hospitalization status revealed that hospitalized patients with low SES had a higher risk of experiencing post-COVID-19 cardiovascular symptoms (HR = 1.96; 95 CI, 1.23–3.12, p = 0.004), while in non-hospitalized, low SES patients a lower risk was observed (HR = 0.82; 95 CI, 0.70–0.97, p = 0.017). No significant association was found between SES and other post-COVID-19 symptoms including general and respiratory symptoms.

**Conclusions:**

We concluded an association between higher SES and increased post-COVID-19 cardiovascular symptoms. Low SES was associated with higher risk of post-COVID-19 cardiovascular symptoms in hospitalized patients, while in non-hospitalized, it was associated with a lower risk.

## Highlights

• Low socioeconomic status (SES) increased the risk of long COVID-19 cardiovascular symptoms in the whole population.
• Hospitalized patients with low SES had a higher risk of long COVID-19 cardiovascular symptoms.
• Non-hospitalized patients with low SES had a lower risk of long COVID-19 cardiovascular symptoms.
• SES was not associated with long COVID-19 general and respiratory symptoms.

## 1 Introduction

The Centers for Disease Control and Prevention (CDC) have introduced the term “Post-Acute Sequelae of COVID-19″ or post-COVID to describe symptoms associated to COVID-19 that persist beyond four weeks (Centers for Disease Control and Prevention, 2021). These persistent symptoms commonly include fatigue, dyspnea, depression, insomnia, anxiety, and memory loss (Han et al., 2022). The prevalence of persistent symptoms is reported as high as 87 % and 50 % among hospitalized and non-hospitalized patients, respectively (Carfì et al., 2020, Armange et al., 2021, Xie et al., 2021).

The persistence of COVID-19 symptoms beyond the acute phase increases healthcare utilization and financial burden for both patients and the healthcare system (González-Hermosillo et al., 2021). Patients with low socioeconomic status (SES) may not seek advanced care due to increased costs and lack of health insurance. Additionally, individuals from lower SES circumstances who have experienced a lifetime of adversity are at an increased risk of having one or more health conditions (Berger et al., 2021), making them a high-risk population for adverse outcomes caused by COVID-19 (Langenberg et al., 2006). Previous studies have shown the association between lower SES and increased vulnerability, hospital admission, morbidity, and worsening outcomes of COVID-19 (Clouston et al., 2021, de Lusignan et al., 2020, Bambra et al., 2020, Holuka et al., 2020), though a number of studies reported higher rates of COVID-19 infection and mortality in high-income areas (Abedi et al., 2021, Khanijahani and Tomassoni, 2021).

Nevertheless, there is a limited literature presenting the relationship between SES and post-COVID symptoms. The main objective of this study was to determine whether SES is associated with post-COVID-19 symptoms among a population of hospitalized and non-hospitalized patients with COVID-19,

## 2 Materials and methods

### 2.1 Study design and population

The Isfahan COVID Cohort (ICC) study is a 5-year longitudinal cohort study started in 2020. The design and methodology of the study were previously described in greater details (Sarrafzadegan et al., 2022). In brief, ICC aimed to evaluate the short-term and persistence comorbidities, their potential risk factors, and mortality rate of patients with COVID-19. ICC included patients who were hospitalized with moderate and severe symptoms of COVID-19 (World Health Organization, 2020). In addition, we recruited patients who were registered in health centers because of experiencing any mild symptoms of COVID-19 or exposing to someone with COVID-19 and were asymptomatic despite having a positive test result of RT-PCR for coronavirus. Therefore, the registered hospitalized and non-hospitalized patients with COVID-19 were invited to health centers located in the urban and rural areas in Isfahan. Hospitalized patients before March 10, 2020, and patients who did not have an RT-PCR test for COVID-19 or those with negative test result of RT-PCR were excluded. This study included 3912 patients who were followed-up for one year. The protocol of the study was approved by the ethics committee of Isfahan University of Medical Sciences (IUMS) (No: **IR.MUI.MED.REC.1399.223).** All the participants provided their informed consent in writing.

### 2.2 Data collection

Various aspects of general characteristics, such as age, sex and smoking status were determined. Moreover, information in terms of pre-existing conditions including coronary heart disease, hypertension, chronic respiratory disease, diabetes mellitus (DM), cancers, and chronic kidney disease (CKD), as well as the medications and risk factors associated with these non-communicable diseases by interviewing patients at health centers using standardized questionnaire. Psychological factors were evaluated using validated Persian version of hospital anxiety and depression scale (Montazeri et al., 2003) and perceived stress scale (Nouri et al., 2018). Additionally, general physical examinations were conducted by GPs at health centers and recorded. Height and weight were measured while participants wore light clothes and no shoes to the nearest 0.5 cm and 0.5 kg, respectively. Body mass index (BMI) was calculated by dividing the weight in kilograms by the square of the height in meters. The measurement of waist circumference (WC) was done by placing the tape at the midpoint between the lower edge of the last rib and the top of the hip bone. On the other hand, the hip circumference was measured at the widest part of the buttocks using a non-elastic tape, with precision to the nearest 0.1 cm. The resting blood pressure was measured two times while participants were seated after 5 min of rest using an OMRON barometer. The average of the two measurements was taken for analysis. The definition of hypertension was based on having a mean systolic blood pressure ≥ 140 mmHg and/or mean diastolic blood pressure ≥ 90 mmHg, or taking antihypertensive medications (Williams et al., 2018).

### 2.3 Follow-up

Patients were contacted every six months and subsequently followed up for one year. If patients developed any new sign or symptom after discharging from hospital, they were referred by general physicians to the COVID-19 clinic and were visited by and internal medicine specialist. General physicians taught and asked all patients to attend this clinic if they developed any problem during the follow-up.

### 2.4 Primary endpoints

Post-COVID-19 was defined as the persistence of the symptoms or development of new symptoms for at least three months after COVID-19 onset. According to organ system involvement, self-reported post-COVID-19 symptoms were categorized into general (abnormal weight loss or gain, generalized fatigue, and loss of appetite), cardiovascular (chest pain, palpitation, lower extremity edema, cold extremities, paresthesia, color change in extremities, and early fatigue with physical activity), and respiratory (continuous and annoying cough, dyspnea, need for oxygen therapy, and wheezing) symptoms.

### 2.5 SES measurement

We evaluated the SES of participants using standard questionnaires including multiple-choice questions (Roohafza et al., 2021). Our online questionnaire has been filled out by the health centers’ trained personnel. These questionnaires encompassed several indicators, including educational level, occupation, house room number, the number of trips taken, and using notebooks, laptop, or tablet in the house. The level of education was classified as low (≤5 years), medium (González-Hermosillo et al., 2021, Berger et al., 2021, Langenberg et al., 2006, Clouston et al., 2021, de Lusignan et al., 2020, Bambra et al., 2020, Holuka et al., 2020), and high (≥13 years). Occupation status was divided into governmental employed, self-employed, housewife, retired, unemployed, and student. We used latent class analysis (LCA) to summarize all the variables into one main total SES, and then was divided into two levels, less than median SES as “low SES” and higher than median SES as “high SES.”

### 2.6 Statistical analysis

The baseline characteristics of subgroups were compared via Student’s *t* test and chi-square test. The association between baseline SES with post-COVID-19 symptoms were examined using Cox proportional hazard analysis in separate steps. First, a crude model was performed with no adjustment for potential confounders. In additional analysis, model 1 adjusted for age (year), sex (male/ female), marital status (single/ married/ divorced or widow), region of residency (urban/ rural) and education. Model 2 additionally adjusted for comorbidities including DM, coronary heart disease, CKD, chronic respiratory disease, and cancer, smoking and hypertension (yes/ no). Model 3 additionally adjusted for severity of COVID 19 (hospitalized/ non hospitalized). P < 0.05 was considered statistically significant. All analysis were performed using SPSS version 24.

## 3 Results

A total of 3912 hospitalized and non-hospitalized patients with confirmed SARS-CoV-2 were included in this study, 1798 (45.9 %) of whom were female, and 2598 participants (66.4 %) reported post-COVID-19 symptoms.

Demographics and baseline characteristics of individuals, stratified by the presence or absence of post-COVID-19 symptoms, are presented in Table 1. Patients with post-COVID-19 symptoms were more probably to be older (P < 0.001), females (P < 0.001), less educated (P = 0.024), have higher number of comorbidities (P < 0.001), or higher prevalence of severe COVID-19 cases (P < 0.001), higher BMI (P < 0.001) and WC (P < 0.001), higher depression and anxiety score (P < 0.001), lower SES (P < 0.001) and live in urban (P < 0.001).

**Table 1:** Baseline characteristics of patients with COVID 19 based on post-COVID symptoms occurrence among participants of Isfahan COVID Cohort (ICC) study, from 2020 to 2023.

**Variable**	**Post-COVID N = 2598 (66.4)**	**Non post-COVID** **N = 1314 (33.6)**	**P value**
**Age mean ± SD**	56.4 ± 14.5	53.4 ± 15.1	<0.001
**Gender (Male) n (%)**	1298 (50.0)	816 (62.1)	<0.001
**Smoking status n (%):**			0.015
Smoker	115 (4.4)	85 (6.5)	
Ex-smoker	116 (4.5)	49 (3.7)	
Non smoker	2367 (91.1)	1180 (89.8)	
**Comorbidities n (%):**			
Diabetes	718 (27.6)	261 (19.9)	<0.001
Hypertension	938 (36.1)	345 (26.3)	<0.001
Renal disease	365 (14.0)	131 (10.0)	<0.001
Coronary heart disease	393 (15.1)	143 (10.9)	<0.001
Chronic respiratory disease	310 (11.9)	63 (4.8)	<0.001
Cancer	70 (2.7)	10 (0.8)	<0.001
**Severity of COVID 19n (%):**			<0.001
Hospitalized	2174 (83.7)	974 (74.1)	
Non-hospitalized	424 (16.3)	340 (25.9)	
**Marital status n (%):**			<0.001
Single	134 (5.2)	105 (8.0)	
Married	2196 (84.5)	1110 (84.5)	
Divorced or widow	268 (10.3)	99 (7.5)	
**Educational level n (%):**			0.024
< 6 years	991 (38.1)	447 (34.1)	
6–12 years	994 (38.3)	517 (39.3)	
> 12 years	613 (23.6)	350 (26.6)	
**Region of residency n (%):**			<0.001
Urban	2424 (94.3)	1163 (90.0)	
Rural	146 (5.7)	129 (10.0)	
**BMI mean ± SD**	28.8 ± 4.7	28.1 ± 4.6	<0.001
**WC mean ± SD**	100.1 ± 11.6	98.3 ± 11.4	<0.001
**SBP mean ± SD**	116.4 ± 29.6	115.4 ± 25.3	0.274
**DBP mean ± SD**	78.5 ± 19.6	79.6 ± 18.1	0.098
**Depression score mean ± SD**	4.8 ± 7.1	2.6 ± 5.1	<0.001
**Anxiety score mean ± SD**	5.2 ± 6.5	3.1 ± 4.8	<0.001
**SES score mean ± SD**	6.1 ± 3.0	6.6 ± 3.1	<0.001

BMI: Body mass index; WC: Waist circumference; SBP: Systolic blood pressure; DBP: Diastolic blood pressure; SES: Socioeconomic status.

The prevalence of reported post-COVID-19 symptoms based on SES level and hospitalization status is represented in Table 2. In hospitalized patients, there was a significant difference between low and high SES participants in terms of generalized fatigue (P = 0.041), loss of appetite (P = 0.002), wheezing (P = 0.008), and total respiratory symptoms (P = 0.044). Moreover, a significant difference was observed in total cardiovascular symptoms, lower extremity edema, cold extremities, paresthesia, and color change in extremities between the low and high SES groups in both hospitalized and non-hospitalized participants.

**Table 2:** Self-reported post-COVID occurrence based on disease severity and socioeconomic status among participants of Isfahan COVID Cohort (ICC) study, from 2020 to 2023.

**System**		**Hospitalized** **N = 3148 (80.5)**			**Non-hospitalized** **N = 764 (19.5)**	
	Low SES* 1442 (45.8)	High SES** 1706 (54.2)	**P value**	**Low SES** **419 (54.8)**	**High SES** **345 (45.2)**	**P value**
**General:**						
Total	851 (59.0)	973 (57.0)	0.262	191 (45.6)	145 (42.0)	0.324
Abnormal Weight loss or gain	482 (33.4)	584 (34.2)	0.634	25 (6.0)	27 (7.8)	0.310
Generalized fatigue	706 (49.0)	773 (45.3)	0.041	160 (38.2)	117 (33.9)	0.221
Loss of appetite	373 (25.9)	361 (21.2)	0.002	67 (16.0)	41 (11.9)	0.105
**Cardiovascular**:						
Total	638 (44.2)	694 (40.7)	0.044	81 (19.3)	33 (9.6)	<0.001
Chest pain	94 (6.5)	88 (5.2)	0.103	11 (2.6)	7 (2.0)	0.589
Palpitation	177 (12.3)	173 (10.1)	0.058	18 (4.3)	11 (3.2)	0.425
Lower extremity edema	157 (10.9)	116 (6.8)	<0.001	10 (2.4)	2 (0.6)	0.046
Cold extremities, paresthesia, color change in extremities	249 (17.3)	234 (13.7)	0.006	17 (4.1)	5 (1.4)	0.032
Early fatigue (with physical activity)	529 (36.7)	591 (34.6)	0.233	64 (15.3)	25 (7.2)	0.001
**Respiratory system:**						
Total	381 (26.4)	399 (23.4)	0.049	83 (19.8)	59 (17.1)	0.338
Continuous and annoying cough	111 (7.7)	102 (6.0)	0.056	17 (4.1)	14 (4.1)	1.000
Dyspnea	283 (19.6)	298 (17.5)	0.120	72 (17.2)	38 (11.0)	0.016
Need for oxygen therapy	113 (7.8)	109 (6.4)	0.114	2 (0.5)	1 (0.3)	0.680
Wheezing	93 (6.4)	74 (4.3)	0.008	16 (3.8)	11 (3.2)	0.639

SES: Socioeconomic status.

* Low SES: Less than median socioeconomic score.

** High SES: Higher than median socioeconomic score.

In comparison with the high SES, the low SES was associated with an increased risk of cardiovascular symptom occurrence (HR = 1.34; 95 % CI: 1.19, 1.56; P = 0.004). After multivariable adjustment, the association of low SES with post-COVID-19 cardiovascular symptoms weakened, but remained statistically significant (HR = 1.18; 95 % CI: 1.03–1.36; P = 0.022) in the whole population. Moreover, this significant association was delighted more, after adjustment for the severity of COVID-19 (HR = 1.15; 95 %CI: 1.01–1.31; P = 0.022). However, there was no association between the SES and other post-COVID-19 symptoms **(**Table 3**)**.

**Table 3:** Hazard ratio and 95% confidence interval of post-COVID symptoms occurrence with socioeconomic status among participants of Isfahan COVID Cohort (ICC) study, from 2020 to 2023.

**System**	High SES*	Low SES**	**P value**
**General:**			
Crude	1	1.06 (0.94, 1.21)	0.352
Model 1	1	0.89 (0.78, 1.03)	0.093
Model 2	1	0.86 (0.75, 1.00)	0.053
Model 3	1	0.93 (0.81, 1.08)	0.317
**Cardiovascular**:			
Crude	1	1.34 (1.19, 1.56)	0.003
Model 1	1	1.23 (1.12, 1.40)	0.015
Model 2	1	1.18 (1.03, 1.36)	0.022
Model 3	1	1.15 (1.01, 1.31)	0.039
**Respiratory system:**			
Crude	1	1.15 (0.997, 1.34)	0.056
Model 1	1	1.07 (0.91, 1.25)	0.602
Model 2	1	1.00 (0.84, 1.18)	0.795
Model 3	1	1.04 (0.87, 1.23)	0.814

SES: Socioeconomic status.

Model 1: Adjusted for age (year), sex (male/ female), marital status (single/ married/ divorced or widow) and region of residency (urban/ rural).

Model 2: Additionally adjusted for comorbidities including diabetes mellitus, hypertension, renal disease, coronary heart disease, chronic respiratory disease and cancer (yes/ no).

Model 3: Additionally adjusted for severity of COVID 19 (hospitalized/ non hospitalized).

* Low SES: Less than median socioeconomic score.

** High SES: Higher than median socioeconomic score as reference group.

Post-COVID-19 symptoms were evaluated in relation to SES separately in hospitalized and non-hospitalized patients **(**Table 4**)**. After full potential adjustment, there was a positive association (HR = 1.96; 95 %CI: 1.23–3.12; P = 0.004) and negative association (HR = 0.82; 95 %CI: 0.70–0.97; P = 0.017) between low SES and long cardiovascular COVID-19 symptoms in hospitalized and non-hospitalized patients, respectively. The general and respiratory symptoms did not show significant differences between low SES and high SES groups **(**Table 4**)**.

**Table 4:** Hazard ratio and 95% confidence interval of post-COVID symptoms occurrence with socioeconomic status based on hospitalization among participants of Isfahan COVID Cohort (ICC) study, from 2020 to 2023.

**System**	High SES*	Low SES**	**P value**
**Hospitalized:**			
**General:**			
Crude	1	1.08 (0.94, 1.25)	0.262
Model 1	1	0.93 (0.79, 1.10)	0.347
Model 2	1	0.89 (0.76, 1.05)	0.155
**Cardiovascular**:			
Crude	1	2.27 (1.47, 3.49)	<0.001
Model 1	1	1.98 (1.25, 3.12)	0.003
Model 2	1	1.96 (1.23, 3.12)	0.004
**Respiratory system:**			
Crude	1	1.18 (1.00, 1.38)	0.050
Model 1	1	1.16 (0.96, 1.39)	0.167
Model 2	1	1.08 (0.89, 1.31)	0.536
**Non-hospitalized:**			
**General:**			
Crude	1	1.15 (0.87, 1.54)	0.325
Model 1	1	1.11 (0.81, 1.52)	0.564
Model 2	1	1.13 (0.82, 1.55)	0.488
**Cardiovascular**:			
Crude	1	1.16 (1.00, 1.33)	0.044
Model 1	1	0.90 (0.77, 1.06)	0.187
Model 2	1	0.82 (0.70, 0.97)	0.017
**Respiratory system:**			
Crude	1	1.20 (0.83, 1.73)	0.339
Model 1	1	0.99 (0.66, 1.48)	0.910
Model 2	1	0.96 (0.63, 1.45)	0.784

SES: Socioeconomic status.

Model 1: Adjusted for age (year), sex (male/ female), marital status (single/ married/ divorced or widow) and region of residency (urban/ rural).

Model 2: Additionally adjusted for comorbidities including diabetes mellitus, renal disease, coronary heart disease, chronic respiratory disease and cancer, smoking and hypertension (yes/ no).

* Low SES: Less than median socioeconomic score.

** High SES: Higher than median socioeconomic score as reference group.

## 4 Discussion

We found that there was a negative correlation between SES level and risk of post-COVID-19 cardiovascular symptoms, regardless of the COVID-19 severity. Hospitalized patients with low SES were at a higher risk of experiencing post-COVID-19 cardiovascular symptoms. However, non-hospitalized patients with low SES showed a lower risk of post-COVID-19 cardiovascular symptoms.

The results of this study share a number of similarities with previous research. Durstenfeld et al. showed a significant association between low SES and increased post-COVID-19 symptoms in an online cohort study including 13,305 individuals (Durstenfeld et al., 2023). In a cohort study of 16,091 adults, participants with lower education, lower income, and rural residences were more likely to report post-COVID-19 symptoms (Perlis et al., 2022). A retrospective population-based cohort study revealed the odds of experiencing post-COVID symptoms that persist for four or more weeks were 46 % greater in the most socioeconomically disadvantaged group compared to the least disadvantaged group (Shabnam et al., 2023). Furthermore, an analyses of 10 UK longitudinal study revealed a correlation between higher levels of education and a reduced likelihood of experiencing post-COVID-19 symptoms (Thompson et al., 2022).

In this study, we categorized post-COVID-19 symptoms into general, cardiovascular, and respiratory symptoms, and we found the association of SES with only post-COVID-19 cardiovascular symptoms. Several factors are thought to be participated in pathophysiology of long cardiovascular symptoms following SARS-CoV-2 infection including decreased expression of the angiotensin-converting enzyme 2 receptor, hypoxic damage, blood clotting, and systemic inflammation (Parhizgar et al., 2023, Davis et al., 2023). A *meta*-analysis of 43 studies found that low SES is associated with higher level of systemic inflammatory markers including C-reactive protein and interleukin-6 (Muscatell et al., 2020). In addition, an increasing number of studies have found the higher risk of thrombosis in individual with low SES (Zöller et al., 2012, Isma et al., 2013). Hence, the association of low SES with systemic inflammation and thrombosis may explain the role of SES for predicting post-COVID-19 cardiovascular symptoms. Further studies are required to clarify the mechanisms through which SES is linked with post-COVID-19 cardiovascular symptoms.

Although some studies have investigated the potential risk factors for post-COVID-19 symptoms in non-hospitalized patients (Pływaczewska-Jakubowska et al., 2022, Kisiel et al., 2022), knowledge regarding association of SES with post-COVID-19 symptoms in non-hospitalized patients is still limited. This study suggested the protective role of low SES in predicting post-COVID-19 cardiovascular symptoms in non-hospitalized patients. However, Subramanian et al. found an increased risk of post-COVID-19 symptoms with decreasing SES level in in non-hospitalized adults (Subramanian et al., 2022). These conflicting findings may be attributed to some reasons. Firstly, Subramanian et al. had a large sample size including over 2.4 million participants, leading to the increased statistical power. Secondly, they used indices of multiple deprivation for area deprivation measures of SES, which is a different method that used to measure SES in our study. Besides, while our study found high SES as an associating risk factor of post-COVID-19 cardiovascular symptoms, Subramanian et al. evaluated the association of SES with total post-COVID-19 symptoms.

Further investigations in diverse populations and larger sample sizes are warranted to provide a comprehensive understanding of the role of SES in post-COVID-19 symptomatology among non-hospitalized individuals.

This study had some strengths. This study is a longitudinal cohort, which allows for the assessment of long-term effects and outcomes of COVID-19 symptoms. We collected a wide range of data, including SES, demographics, comorbidities, lifestyle factors, and psychological variables. This comprehensive approach allows for a thorough analysis of the association between SES and post-COVID-19 symptoms, while also considering potential confounding factors. We included both hospitalized and non-hospitalized patients with COVID-19, providing a more comprehensive understanding of the impact of SES on post-COVID symptoms. Moreover, this paper included a substantial sample size of 3912 patients, which increases the statistical power and generalizability of the findings. A number of potential limitations need to be considered. First, the symptoms associated with post-COVID are diverse and our study might not have encompassed the complete spectrum of these symptoms. Second, the assessment of SES and other variables relied on self-reported data, which may introduce reporting bias.

In conclusion, this study found that individuals with lower SES may be at higher risk of developing long-term cardiovascular complications following COVID-19 infection, particularly in hospitalized patients. However, there was no significant correlation between SES and other post-COVID-19 symptoms. These results highlight the importance of considering SES in the evaluation and management of post-COVID-19.

## 5 Disclosure of funding and conflicts of interest

Isfahan COVID Cohort (ICC) study has been supported by grant number 199,093 from Isfahan University of Medical Sciences, grant number RPPH 20–76 from the WHO/ EMR, grant number 996,353 from National Institute of Health Researches of Iran, and grant number 99,008,516 from Iran National Science Foundation (INSF). The authors declared no competing interest.

## CRediT authorship contribution statement

**Mehrdad Rabiee rad:** Writing – review & editing, Writing – original draft. **Mehdi Abbasi:** Writing – original draft. **Emad Salimian:** Writing – original draft. **Matin Norouzi:** Writing – original draft. **Ali Emamjomeh:** Writing – original draft. **Fahimeh Haghighatdoost:** Writing – review & editing, Project administration. **Shirin Mahmoudi:** Writing – review & editing, Validation. **Jamshid Najafian:** Validation, Supervision, Project administration. **Soraya Masoudi:** Writing – review & editing, Validation. **Ghazal Ghasempour Dabaghi:** Writing – review & editing, Writing – original draft. **Noushin Mohammadifard:** Writing – review & editing, Project administration, Methodology, Investigation, Conceptualization. **Nizal Sarrafzadegan:** Supervision, Resources, Project administration, Methodology, Investigation, Data curation, Conceptualization.

## Declaration of competing interest

The authors declare that they have no known competing financial interests or personal relationships that could have appeared to influence the work reported in this paper.

## Data Availability

The authors do not have permission to share data.
